# Sympathetic Denervation and Pharmacological Stimulation of Parasympathetic Nervous System Prevent Pulmonary Vascular Bed Remodeling in Rat Model of Chronic Thromboembolic Pulmonary Hypertension

**DOI:** 10.3390/jcdd10020040

**Published:** 2023-01-23

**Authors:** Andrei A. Karpov, Nikita S. Vachrushev, Leonid A. Shilenko, Sergey S. Smirnov, Nikolay S. Bunenkov, Maxim G. Butskih, Al-Khalim A. Chervaev, Dariya D. Vaulina, Dmitry Yu. Ivkin, Olga M. Moiseeva, Michael M. Galagudza

**Affiliations:** 1 Institute of Experimental Medicine, Almazov National Medical Research Centre, 2 Akkuratova Street, 197341 St. Petersburg, Russia; 2 Department of Experimental Pharmacology, State Federal-Funded Educational Institution of Higher Education, Saint Petersburg State Chemical and Pharmaceutical University of the Ministry of Healthcare of the Russian Federation, 14 Professora Popova Street, 197022 St. Petersburg, Russia; 3 Institute of Molecular Biology and Genetics, Almazov National Medical Research Centre, 2 Akkuratova Street, 197341 St. Petersburg, Russia; 4 Department of Bone Marrow Transplantation, Raisa Gorbacheva Research Institute of Children Oncology, Hematology and Transplantation of Pavlov First Saint Petersburg State Medical University, 6–8 L’va Tolstogo Street, 197022 St. Petersburg, Russia; 5 Department of Pathophysiology with Clinical Pathophysiology Course, Pavlov First Saint Petersburg State Medical University, 6–8 L’va Tolstogo Street, 197022 St. Petersburg, Russia; 6 Institute of Heart and Vessels, Almazov National Medical Research Centre, 2 Akkuratova Street, 197022 St. Petersburg, Russia

**Keywords:** pulmonary hypertension, chronic thromboembolic pulmonary hypertension, autonomic nervous system, sympathetic denervation, vagus denervation, pyridostigmine, rodent model, pulmonary vascular remodeling

## Abstract

Chronic thromboembolic pulmonary hypertension (CTEPH) develops in 1.5–2.0% of patients experiencing pulmonary embolism (PE) and is characterized by stable pulmonary artery obstruction, heart failure, and poor prognosis. Little is known about involvement of autonomic nervous system (ANS) in the mechanisms of CTEPH. This study was aimed at evaluation of the effect of vagal and sympathetic denervation, as well as stimulation of the parasympathetic nervous system, on the outcomes of CTEPH in rats. CTEPH was induced by multiple intravenous injections of alginate microspheres. Sympathetic and vagal denervation was performed using unilateral surgical ablation of the stellate ganglion and vagotomy, respectively. Stimulation of the parasympathetic nervous system was carried out by administering pyridostigmine. The effect of neuromodulatory effects was assessed in terms of hemodynamics, histology, and gene expression. The results demonstrated the key role of ANS in the development of CTEPH. Sympathetic denervation as well as parasympathetic stimulation resulted in attenuated pulmonary vascular remodeling. These salutary changes were associated with altered MMP2 and TIMP1 expression in the lung and decreased FGFb level in the blood. Unilateral vagotomy had no effect on physiological and morphological outcomes of the study. The data obtained contribute to the identification of new therapeutic targets for CTEPH treatment.

## 1. Introduction

Pulmonary hypertension (PH) is defined as a complex of symptoms that develops in a number of diseases of different etiology. Irrespective of the cause, PH is characterized by typical structural remodeling of the pulmonary vascular bed, which leads to disease progression, right ventricular heart failure, and poor outcome [1]. Chronic thromboembolic pulmonary hypertension (CTEPH) is a rare form of PH which arises in some patients experiencing pulmonary embolism (PE) due to persistent vascular obstruction and microvascular involvement [2]. The incidence of CTEPH varies from 3 to 30 cases per 1,000,000 individuals [3]. According to prospective observational trials, the cumulative incidence of CTEPH varied from 0.1 to 9.1% at two years after acute PE [2]. Such a wide range might stem from the non-specific symptoms of CTEPH and difficulties in distinguishing recurrent episodes of acute PE and established CTEPH [4]. The key event in CTEPH pathogenesis is a fibrotic thrombus transformation resulting in prolonged obstruction of relatively large pulmonary artery branches, which provides a major stimulus for remodeling of distal vasculature [5]. It should be noted, however, that pulmonary vascular obstruction is transient in the majority of PE cases, which underlines the importance of other, yet poorly defined mechanisms of PE-to-CTEPH conversion. For instance, there is some evidence that CTEPH patient-derived endothelial cells isolated from pulmonary thromboendarterectomy specimens demonstrated increased proliferation, decreased angiogenic response, and reduced apoptosis rate. Collectively, these characteristics might contribute to uncontrolled endothelial cell proliferation and formation of hyperplastic intima, which, in turn, facilitates stenosis progression and promotes subsequent vascular remodeling [6]. Aseptic inflammation is also one of the mechanisms of CTEPH development as in situ thrombi formation via activation of proinflammatory factors in pulmonary endothelial cells [7,8].

CTEPH is the only type of PH which is amenable to surgical treatment by means of pulmonary thromboendarterectomy and/or balloon angioplasty, which are becoming standard-of-care around the world [9]. Nevertheless, between 10 and 50% patients with CTEPH are not candidates for endovascular treatment, which stimulates the intense search for new therapeutic targets in CTEPH [10]. Moreover, up to 50% patients subjected to pulmonary thromboendarterectomy tend to have persistent PH or PH recurrence [10]. The persistence of PH after thromboendarterectomy be accounted for by incomplete removal of organized thrombi from the distal parts of vascular tree and/or comorbid vascular lesions.

While considerable efforts have been directed at establishment of the role of adrenergic innervation in the process of systemic vascular remodeling, especially in arterial hypertension, the involvement of sympathetic nervous system (SNS) in fibrotic remodeling of pulmonary vessels and resulting right ventricular failure is much less studied [11]. Pulmonary vessels are innervated by sympathetic, parasympathetic and sensory neuronal fibers. It is known that acute elevation of pulmonary vascular resistance (PVR) might be mediated by stimulation of alpha1-adrenoreceptors localized on vascular smooth muscle cells of pulmonary vessels [12]. Microneurography studies showed the increased tone of sympathetic nerves in patients with pulmonary arterial hypertension (PAH) versus healthy individuals, a finding further supported by elevated whole-body norepinephrine spillover in this category of patients [13,14]. In general, parasympathetic nervous system (PSNS) exerts an opposite effect on vascular smooth muscle cell tone and vascular resistance through NO-dependent mechanisms [15]. Given that adrenergic nerve fibers in humans spread deeply into the lungs innervating the pulmonary arterioles with diameter of more than 50 µm [15], it is conceivable that increased sympathetic activity is one of the major factors contributing to establishment and progression of CTEPH. This idea has been explored in the clinical studies addressing the effect of surgical pulmonary artery (PA) denervation on elevated pressure in the PA [16,17]. In particular, pulmonary artery denervation was investigated in patients with PAH in several clinical trials, demonstrating beneficial effects on PVR and results of six-minute walk test (SMWT) [18,19,20,21]. It should be noted, however, that despite continuous technical improvement, renal sympathetic denervation in patients with resistant arterial hypertension resulted in a limited overall benefit, warranting further search of predictors of blood pressure response to the procedure [22,23]. At present, there are few reports on the effectiveness of pulmonary artery denervation in patients with CTEPH. For instance, Romanov et al. have shown that pulmonary artery denervation after pulmonary thromboendarterectomy resulted in greater reduction in pulmonary vascular resistance than therapy with soluble guanylate cyclase stimulator riociguat at one-year of follow up [24]. Additionally, there is a clinical case presenting female patient with CTEPH subjected to pulmonary thromboextraction/thrombectomy followed by radiofrequency ablation of pulmonary artery [25]. Pulmonary artery denervation resulted in a reduction of pressure by ~ 10 mm Hg, which confirms the role of vascular spasm as a potentially reversible contributor to elevated PVR. There are, however, significant gaps in our understanding of the role of autonomic nervous system in the mechanisms of CTEPH. Therefore, in this study we aimed to test the effects of sympathetic denervation, vagal denervation, and pharmacological parasympathetic nervous system stimulation on physiological and morphological outcomes in rat model of CTEPH. 

## 2. Materials and Methods

### 2.1. Animals and Ethics

Experiments were performed on 61 male Wistar rats (238 ± 21 g body weight). All animals were maintained in standardized conditions, had free access to tap water and were fed with standard rat diet ad libitum. Experimental protocol was performed according to the NIH “Guide for the Care and Use of Laboratory Animals” and approved by Local Ethics Committee. The experiments complied with the ARRIVE guidelines (http://www.nc3rs.org/ARRIVE, accessed on 10 February 2021).

### 2.2. CTEPH Model

CTEPH was induced in animals by means of repetitive embolization of distal branches of pulmonary artery with partially biodegradable alginate microspheres (MS) of 160–200 μm in diameter. MSs were produced from ultrapure alginate natrium (Sigma-Aldrich, St. Louis, MO, USA) using 2% barium chloride as a stabilizer with electrostatic encapsulator (B-390, Buchi, Flawil, Switzerland). All MSs were produced in sterile conditions. 50 μL of MSs by volume were suspended in 1 mL of 0.9% NaCl solution and administered via the tail vein 8 times separated by 4-day intervals [26,27]. Ten rats received injections of 0.9% NaCl solution instead of MSs suspension, thus forming the group of intact animals (INT).

### 2.3. Study Design

**Figure 1:**
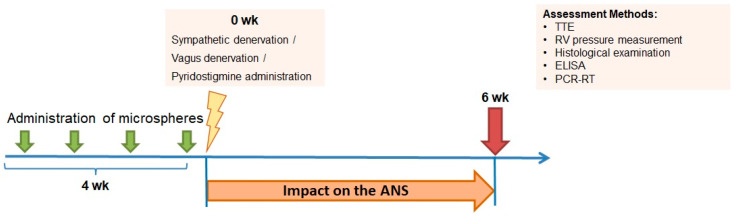
Study design. After induction of CTEPH by intravenous administration (four times, weekly) of alginate microspheres, the animals were randomized into 6 groups, including intact animals, CTEPH, CTEPH + SD, CTEPH + VD, and CTEPH + PS. CTEPH—chronic thromboembolic pulmonary hypertension, CTEPH + SD—CTEPH + sympathetic denervation, CTEPH + VD—CTEPH + vagus denervation, CTEPH + PS—CTEPH + pyridostigmine. Six weeks after surgery, the end-points were analyzed, including echocardiography, invasive hemodynamic measurements, histopathological evaluation of the lung vessels, circulating biomarkers, and gene expression in the lung and heart. TTE—transthoracic echocardiography, ELISA—enzyme linked immunosorbent assay, PCR-RT—real-time polymerase chain reaction, wk—week. ANS—autonomic nervous system.

On the next day after last MSs or vehicle administration, all survived animals were randomly divided into the following groups (Figure 1):

1. Healthy animals (INT)—sham surgery has been performed in these animals which received vehicle only;
2. CTEPH—sham surgery has been performed in these animals with CTEPH;
3. CTEPH + Sympathetic Denervation (CTEPH + SD)—the animals with CTEPH were subjected to unilateral surgical sympathetic denervation;
4. CTEPH + Vagal Denervation (CTEPH + VD)—the animals with CTEPH were subjected to unilateral surgical vagal denervation;
5. CTEPH + Pyridostigmine (CTEPH + PS)—the animals with CTEPH were treated with reversible cholinesterase inhibitor pyridostigmine.

Six weeks after surgery, echocardiography was performed in all animals. Then, hemodynamic measurements were carried out in anesthetized animals, followed by blood sampling, euthanasia and heart and lung harvesting for histological and molecular biology studies.

### 2.4. Surgical Interventions: Sympathetic and Vagal Denervation

All surgical interventions were performed under isoflurane by a SomnoSuite Low-Flow Anesthesia System for gas anesthesia (Kent Scientific, Torrington, CT, USA). Core body temperature was maintained at 37.0 ± 0.5 °C by a feedback-controlled heating pad (TCAT-2LV controller; Physitemp Instruments Inc., Clifton, NJ, USA). Unilateral sympathetic denervation was performed by means of right stellate ganglion destruction. The right stellate ganglion was accessed through the linear cutaneous incision along the anterior axillary line, followed by thermal coagulation of the ganglion and wound closure. The animals were recovered in thermostatic chamber. Denervation was verified by means of ptosis on the side of procedure. Sham-operation included incision and stellate ganglion exposure without destruction. Unilateral vagal denervation was performed by right-sided vagotomy at the level of C4-C5. Cervical portion of vagus nerve was identified within the neurovascular bundle lying medially to the right sternocleidomastoideus muscle after midline skin incision. Sham procedure included only vagus isolation from surrounding tissues. After manipulations on vagus nerve, the skin was sutured (Vicryl 4/0, Ethicon, Cincinnaty, OH, USA).

### 2.5. Pharmacological Stimulation of Parasympathetic Nervous System

Total parasympathetic activity has been increased by means of treating the animals with cholinesterase inhibitor pyridostigmine at a daily dose of 40 mg/kg [28]. The drug was administered intragastrically each day for 6 weeks using commercially available polypropylene feeding tube (Figure 1).

### 2.6. Transthoracic Echocardiography (TTE)

TTE was performed in all animals at 6 weeks after surgery. To perform the study, a high-resolution ultrasound unit (MyLab One Touch SL 3116, Esaote, Genoa, Italy) with a vascular linear probe (frequency: 13 MHz, scanning depth: 2 cm) was used. During TTE, the animals were anesthetized via isoflurane inhalation using a SomnoSuite Low-Flow Anesthesia System for gas anesthesia (Kent Scientific, Torrington, CT, USA) and placed on a heated table (TCAT-2LV controller, Physitemp Instruments Inc., Clifton, NJ, USA) in the supine position. The following parameters were evaluated: pulmonary artery diameter (PAD, mm), right ventricle outflow tract diameter (RVOT, mm), tricuspid annular plane systolic excursion (TAPSE, mm) and fractional shortening (FS, %).

### 2.7. Invasive Hemodynamic Measurements

Before invasive hemodynamic measurements, the animals were anesthetized with intramuscular administration of Zoletil (Virbac, Carros, France) and Xylazine (2%, Interchemie Werken de Adelaar BV, Venray, The Netherlands). Artificial lung ventilation was carried out through tracheal intubation using a ventilator (SAR-830/AP, CWE Inc., Ardmore, PA, USA). The following parameters of artificial lung ventilation were used: respiratory rate: 60–70/min; respiratory capacity: 3 mL/100 g of body weight. Cardiac output (CO) was measured by a volume flow sensor (TS420 Perivascular Flow Module, Transonic, Ithaca, NY, USA) using Doppler flowmetry probe placed on ascending aorta (Transonic, Ithaca, NY, USA). The sensor was inserted into the ascending part of the aorta. Mean blood pressure (mean BP) was monitored in the common carotid artery by the PhysExp Mini recorder (Cardioprotect Ltd., St. Petersburg, Russia). Heart rate was derived from pressure curve. Right ventricular systolic pressure (RVSP) was measured through a needle inserted into the right ventricular cavity through the apex and connected to PhysExp Mini pressure recorder (Cardioprotect Ltd., St. Petersburg, Russia).

### 2.8. Histological Examination

The animals were euthanized with potassium chloride given intravenously under deep isoflurane anesthesia (10% solution, 1 mL, i.v.). During autopsy, the lower lobe of right lung was harvested for morphological studies. The lobe was excised and cut into 4 equal slices. Lung samples were fixed in buffered 10% paraformaldehyde, embedded in paraffin, cut into 3–5-μm sections and stained with hematoxylin and eosin (H&E). After H&E staining, the slides were observed and photos were taken using an optical microscope (Eclipse Ni-U, Nikon, Tokyo, Japan) at 5×–40×. Morphometry was performed using commercially available software (Nis Elements Br4, Nikon, Tokyo, Japan). The mean outer diameter and index of hypertrophy were calculated in all visualized pulmonary artery branches. The index of hypertrophy was defined as the ratio of vessel wall area to the entire cross-sectional area of the vessel (wall plus lumen). The index of hypertrophy was separately calculated in three categories of vessels according to their diameter: <100 μm, 100–199 μm, ≥200 μm. The slides were analyzed by a pathologist blinded to the treatment mode used for each group.

For morphological study of right ventricle, heart was cut into three equal transverse slices just below the left atrial appendage. Slices with 4–5 μm in thickness were stained with hematoxylin and eosin. As assessment criteria of right ventricle remodeling ratio of the right ventricle area versus left ventricle area was used.

### 2.9. Enzyme-Linked Immunosorbent Assay

Serum concentration of profibrotic factors such as transforming growth factor beta (TGFβ) and basic fibroblast growth factor (FGFb) was performed using commercial assays for enzyme-linked immunosorbent assay (ELISA) (Abcam, Cambridge, UK). The obtained results were analyzed spectrophotometrically (Model 680, Bio-Rad, Hercules, CA, USA).

### 2.10. Gene Expression Analysis

Lung and cardiac samples were obtained post mortem, followed by immediate freezing in liquid nitrogen for transportation. Furter storage was at −80 °C. Frozen samples were homogenized with bead mill (TissueLyzer, QIAGEN, Hilden, Germany) during 5 min in Extract RNA reagent medium (Evrogen, Moscow, Russia). Quality and quantity of RNA were confirmed with spectrophotometry (NanoDrop 3300, Thermo Fisher Scientific, Waltham, MA, USA) and agarose gel electrophoresis. Extracted mRNA was reverse transcribed to get cDNA from RNA matrices. For this goal, reverse transcription mechanism was used with Random (dN)10-primer (Evrogen) and MMLV RT kit (Evrogen). The amplification of the newly synthesized cDNA was carried out by the standard PCR procedures (QuantStudio™ 5 Real-Time PCR System, Applied Biosystems, Waltham, MA, USA) in 384-well plate in the following conditions: 95 °C during 10 min, 95 °C during 15 s and 60 °C during 1 min. $2^{-△△C_{T}}$ method with normalization to reference gene (GAPDH) expression was used (Table 1).

### 2.11. Statistical Analysis

Results are presented as mean ± standard deviation. Statistical analysis was performed by Statistica 10. Given the small sample sizes and non-normal distribution, the differences were analyzed using non-parametric tests. The Kruskal–Wallis test was used to determine the overall differences between groups in all tested end-points. Further, pairwise comparisons between groups were performed using the non-parametric Mann–Whitney U test. Differences were considered significant when *p* < 0.05.

## 3. Results

### 3.1. Mortality and Exclusions

**Figure 2:**
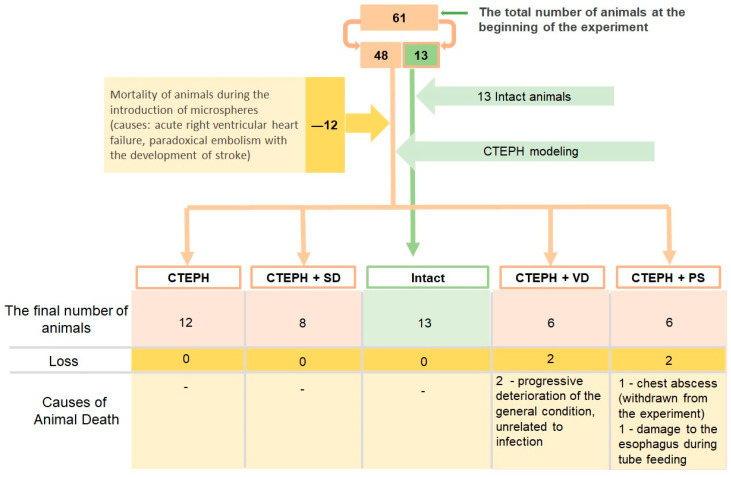
This is a figure. Schemes follow the same formatting. Flow chart of the experimental procedures showing animal mortality and exclusions. CTEPH—chronic thromboembolic pulmonary hypertension, CTEPH + SD—CTEPH + sympathetic denervation, CTEPH + VD—CTEPH + vagus denervation, CTEPH + PS—CTEPH + pyridostigmine.

MSs administrations, especially the initial one, caused death in 12 animals. The main causes of death were acute right ventricular heart failure (obstructive shock) and paradoxical embolism associated with severe neurological deterioration. A detailed scheme of animal losses during experiment is shown in Figure 2.

There were no significant intergroup differences in echocardiographic parameters (Table 2).

### 3.2. Hemodynamic Parameters

**Figure 3:**
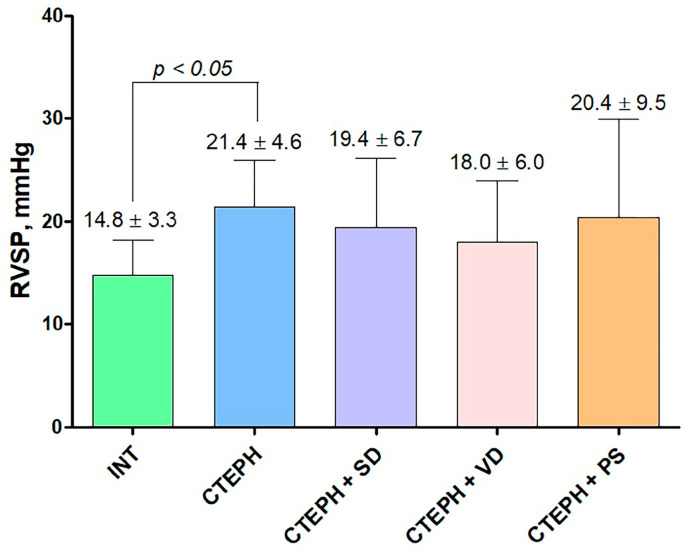
Right ventricular systolic pressure (RVSP) measured by RV apical puncture 6 weeks after microspheres administration. INT—intact animals (n = 13), CTEPH—chronic thromboembolic pulmonary hypertension (n = 11), CTEPH + SD—CTEPH + sympathetic denervation (n = 8), CTEPH + VD—CTEPH + vagus denervation (n = 5), CTEPH + PS—CTEPH + pyridostigmine (n = 5).

RV pressure measurement at 6 weeks after surgery demonstrated that RVSP was higher in CTEPH group versus INT group (Figure 3). There were no significant differences between groups in other hemodynamic parameters (Table 3).

### 3.3. Histological Examination

**Figure 4:**
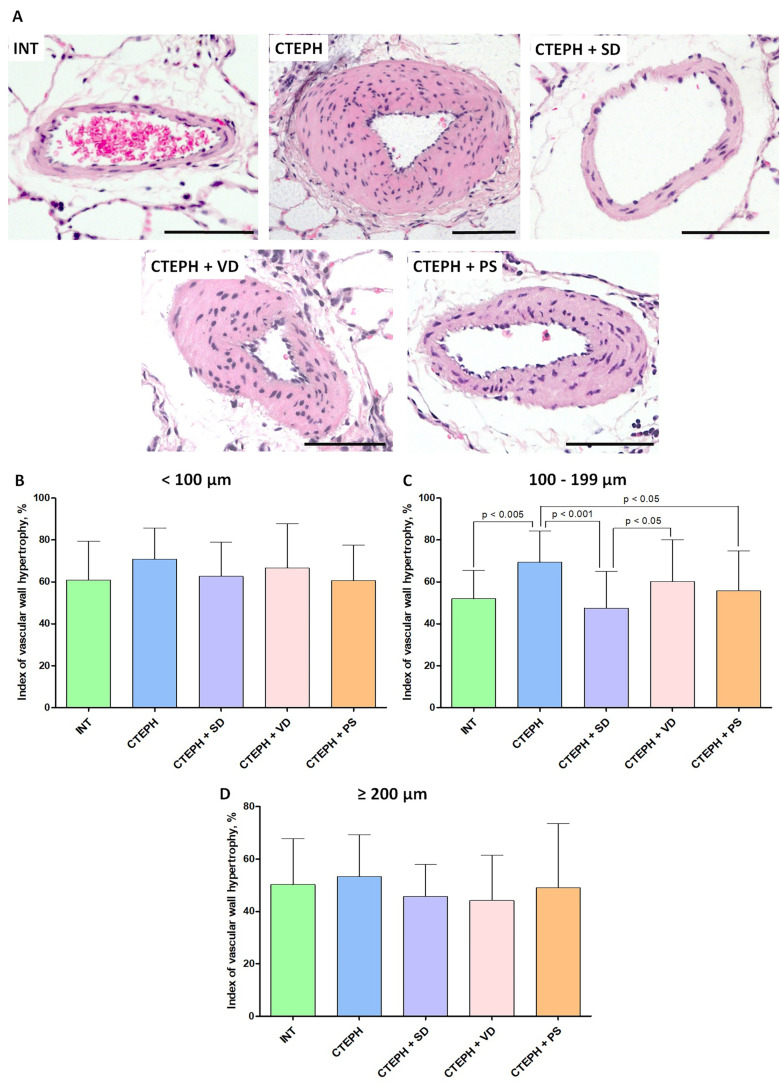
Histological examination of the lung vessels. (**A**)—representative microphotographs of the branches of the pulmonary artery in studied groups, staining: hematoxylin–eosin, scale bar: 100 μm. (**B**–**D**)—Index of hypertrophy in pulmonary artery branches with different diameters at the end of 6-week follow up period after microspheres administration. (**B**)—outer diameter of vessel < 100 μm, (**C**)—outer diameter of vessel 100–199 μm, (**D**)—outer diameter of vessel ≥ 200 μm, INT—intact animals (n = 7), CTEPH—chronic thromboembolic pulmonary hypertension (n = 6), CTEPH + SD—CTEPH + sympathetic denervation (n = 6), CTEPH + VD—CTEPH + vagus denervation (n = 6), CTEPH + PS—CTEPH + pyridostigmine (n = 6).

In total, 439 microscopic images of pulmonary arteries have been analyzed. Among those, there were 164 arteries of <100 µm in diameter, 203 arteries with diameter from 100 to 199 µm, and 72 arteries with diameter of ≥200 µm (Figure 4). Occasionally, individual MSs were visualized in the vascular lumen. The greatest differences among groups were identified in the subset of arteries from 100 to 199 µm (Figure 4B). In particular, the index of hypertrophy was significantly higher in CTEPH group versus INT group (69.3 ± 14.9 vs. 51.9 ± 13.5, respectively; *p* < 0.005). Both sympathetic denervation and pyridostigmine administration resulted in significantly smaller index of hypertrophy (47.5 ± 17.5 and 55.6 ± 19.2, *p* < 0.05 in comparison with CTEPH). Vagal denervation had no effect on the index of hypertrophy (60.2 ± 19.9).

### 3.4. Enzyme-Linked Immunosorbent Assay

**Figure 5:**
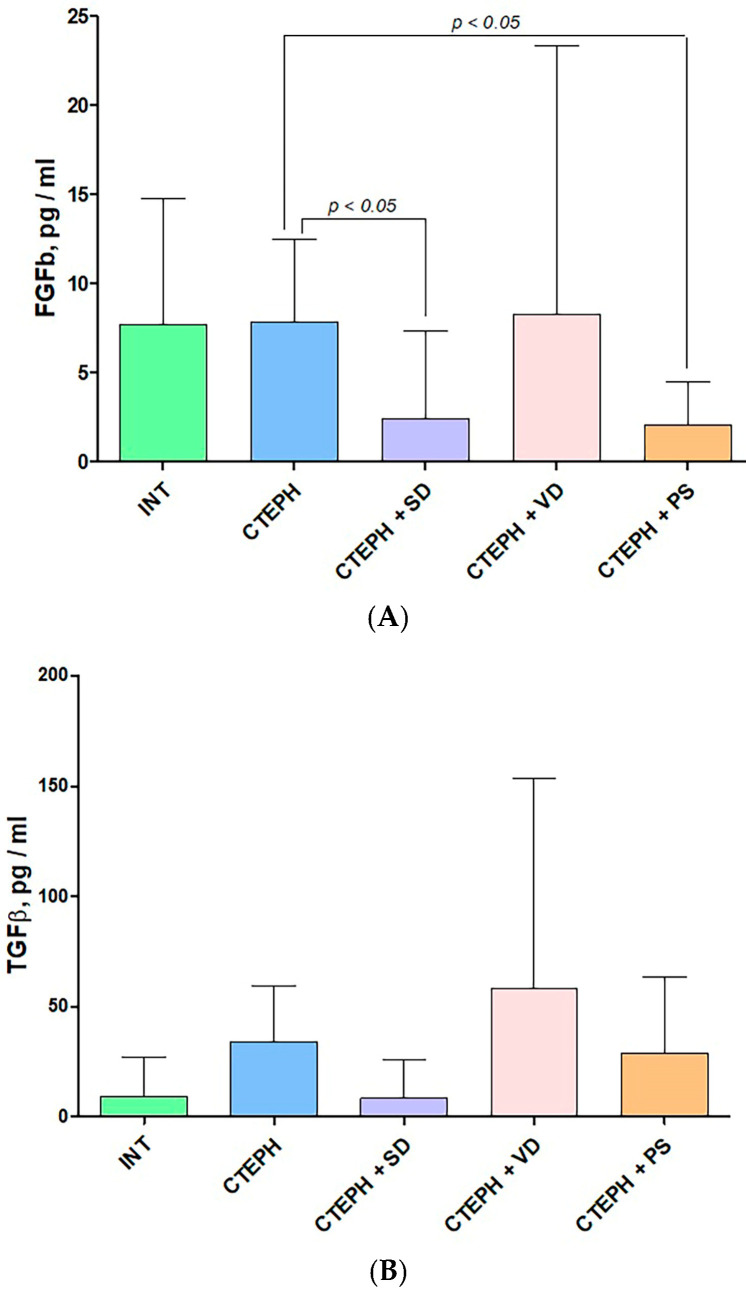
The plasma levels of FGFb (**A**) and TGFβ (**B**) in experimental groups. INT—intact animals (n = 6), CTEPH—chronic thromboembolic pulmonary hypertension (n = 6), CTEPH + SD—CTEPH + sympathetic denervation (n = 6), CTEPH + VD—CTEPH + vagus denervation (n = 6), CTEPH + PS—CTEPH + pyridostigmine (n = 6), FGFb—basic fibroblast growth factor, TGFβ—transforming growth factor beta.

When analyzing the plasma levels of FGFb and TGFβ, we found significantly reduced concentration of FGFb in CTEPH + SD and CTEPH + PS groups in comparison to CTEPH group, although FGFb level was not different in CTEPH and INT groups (CTEPH 7.88 ± 4.60, CTEPH + SD 2.54 ± −4.91, CTEPH + PS 2.03 ± 2.44 pg/mL, *p* > 0.05, Figure 5A). TGFβ level was not different among experimental groups (Figure 5B).

### 3.5. Gene Expression Analysis

**Figure 6:**
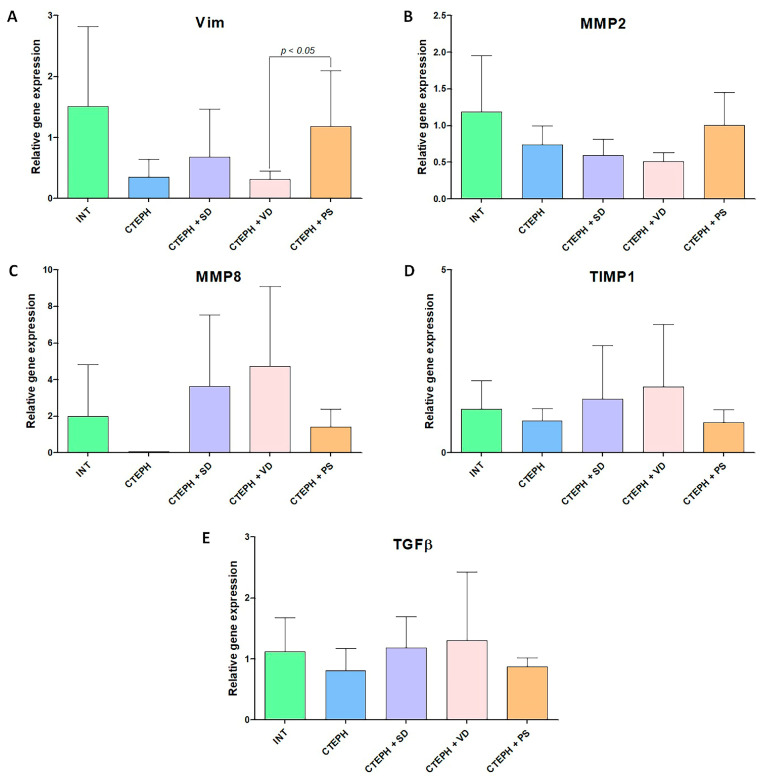
Analysis of expression of profibrotic factors in myocardial tissue with different variants of activity of vegetative nervous system. (**A**)—relative gene expression of Vim, (**B**)—relative gene expression of MMP2, (**C**)—relative gene expression of MMP8, (**D**)—relative gene expression of TIMP1, (**E**)—relative gene expression of TGFβ. INT—intact animals (n = 8), CTEPH—chronic thromboembolic pulmonary hypertension (n = 5), CTEPH + SD—CTEPH + sympathetic denervation (n = 8), CTEPH + VD—CTEPH + vagus denervation (n = 6), CTEPH + PS—CTEPH + pyridostigmine (n = 5). Vim—vimentin, MMP2—matrix metalloproteinase-2, MMP8—matrix metalloproteinase-8, TIMP1—tissue inhibitor of metalloprotease-1, TGFβ—transforming growth factor beta.

Fibrotic gene expression in the right ventricular myocardium and lung. According to RT-PCR expression analysis in the myocardium, VIM expression was significantly higher in CTEPH + PS group compared to CTEPH + VD group (Figure 6A). Relative expression of other genes in the right ventricle was not different between groups (Figure 6).

**Figure 7:**
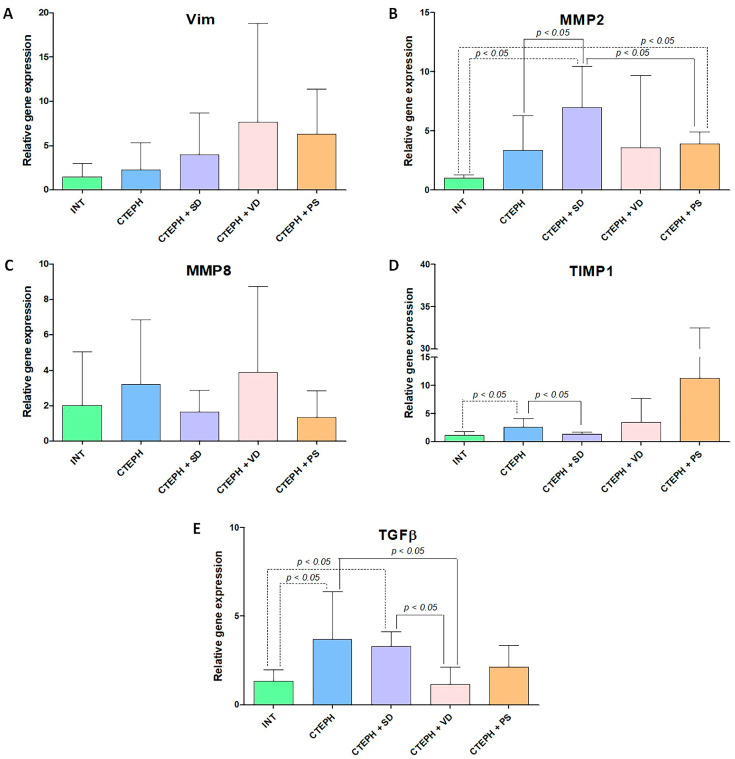
Analysis of expression of profibrotic factors in lung tissue in different experimental groups. (**A**)—relative gene expression of Vim, (**B**)—relative gene expression of MMP2, (**C**)—relative gene expression of MMP8, (**D**)—relative gene expression of TIMP1, (**E**)—relative gene expression of TGFβ. INT—intact animals (n = 8), CTEPH—chronic thromboembolic pulmonary hypertension (n = 11), CTEPH + SD—CTEPH + sympathetic denervation (n = 8), CTEPH + VD—CTEPH + vagus denervation (n = 6), CTEPH + PS—CTEPH + pyridostigmine (n = 5). Vim—vimentin, MMP2—matrix metalloproteinase-2, MMP8 - matrix metalloproteinase-8, TIMP1—tissue inhibitor of metalloprotease-1, TGFβ—transforming growth factor beta.

There were significant inter-group differences in matrix metalloproteinase (MMP) 2, tissue inhibitor of metalloprotease-1 (TIMP1), and TGFβ expression in the lung (Figure 7).

MMP2 gene expression was significantly lower in INT group than in CTEPH + SD and CTEPH + PS groups (*p* < 0.05, Figure 7B). Further, MMP2 expression was higher in CTEPH + SD group versus CTEPH as well as CTEPH + PS groups (Figure 7B). TIMP1 gene expression was significantly higher in CTEPH group than in CTEPH + SD and INT groups (Figure 7D). TGFβ expression was higher in CTEPH and CTEPH + SD groups versus INT group (Figure 7E). However, TGFβ expression was reduced in CTEPH + VD group in comparison with both CTEPH and CTEPH + SD groups (Figure 7E).

## 4. Discussion

The results obtained demonstrate that surgical sympathetic denervation as well as pharmacologically mediated increase in cholinergic transmission resulted in reduced remodeling of 100–200 μm pulmonary arteries after repetitive episodes of PA embolization mimicking CTEPH development in our animal model. Unilateral vagotomy had no effect on physiological and morphological outcomes of the study. In this study, we used rat CTEPH model based on repetitive intravenous administration of partially biodegradable alginate microspheres [26,27]. In this model, progressive increase in embolic load followed by aseptic inflammation of vascular wall and activated fibrotic signaling results in a typical pattern of pulmonary vascular remodeling associated with residual microvascular obstruction. In the group subjected to stellate ganglion ablation, we have convincingly demonstrated reduced remodeling of vascular wall in 100–200 μm pulmonary arteries. These results correspond well to previously published data on the effects of sympathetic denervation in pulmonary arterial hypertension models [29].

Pulmonary vascular remodeling in PH, CTEPH including, is governed by complex molecular mechanisms. There are three main cell types engaged in the pathogenesis of vascular wall remodeling in PH—endothelial cells, vascular smooth muscle cells, and fibroblasts. The endothelium plays initializing and therefore crucial role in the regulation of both hypertrophy and fibrosis of the vascular wall in PH. This has been demonstrated in a number of previous studies [30,31]. Reduced endothelial production of nitric oxide and prostacyclin as well as increased generation of thromboxane A_2_ and endothelin-1 have been demonstrated to be involved in a process of smooth muscle cell hypertrophy [32]. Endothelin-1 has also been implicated in stimulation of diffuse vascular wall fibrosis [33]. In addition, the role of such profibrotic factors as TGFβ, FGFb, and MMPs in the process of vascular fibrosis has been discussed in the literature [34,35,36].

Our gene expression profiling showed that MMP2 expression has been significantly increased after sympathetic denervation, which was accompanied by appreciable decrease in TIMP1 expression in the same group versus CTEPH. Taking into account a plausible link between MMP2/TIMP1 activity ratio and fibrotic remodeling in CTEPH it might be speculated that altered expression of these target genes is in fact responsible for beneficial effect of sympathetic denervation [37]. In addition, our analysis of serum levels of paracrine factors involved in cardiac and pulmonary remodeling in CTEPH showed that sympathetic denervation resulted in decreased concentration of FGFb in the blood compared to CTEPH group. Given the important role of FGFb in the mechanisms of CTEPH development, these data provide important clues regarding the molecular mechanisms of efficacy of sympathetic denervation in CTEPH. Pharmacological stimulation of parasympathetic nervous system with pyridostigmine resulted in effects generally similar to those elicited by sympathetic denervation. In particular, the index of hypertrophy was significantly smaller in CTEPH + P group versus CTEPH group. To the best of our knowledge, the beneficial effects of parasympathetic stimulation on CTEPH pathomechanisms have been demonstrated in this study for the first time. However, these data fit well to the previously published evidence for salutary effects of enhanced cholinergic transmission in the models of PAH [28]. Our gene expression study has unequivocally showed that myocardial VIM expression has been restored to control values in CTEPH + P group, providing additional proof for close relationship between VIM expression and parasympathetic tone. Taking into account the key role of vimentin in a process of fibrotic remodeling, the data on its increased expression in pyridostigmine group seemingly contradict morphological findings on attenuated remodeling, which requires further investigation [38]. Similarly, to the changes observed in CTEPH + SE group, pyridostigmine administration has resulted in significantly reduced level of FGFb in the blood, which might at least partially explain the effectiveness of the enhanced parasympathetic activity in terms of CTEPH. At the same time, vagal denervation failed to influence the hemodynamic and morphological end-points of the study. Interestingly, vagal denervation resulted in a significantly decreased expression of TGF-β in the lung tissue in comparison with CTEPH and CTEPH + SD groups. Again, these findings are not supported by the morphological data showing the lack of anti-remodeling effect of vagotomy, raising the question about the pathogenetic role of TGF-β in the process of fibrosis perpetuation in the pulmonary circulatory bed versus systemic circulation [28]. It should be noted that, despite clear amelioration of pulmonary vessel remodeling due to sympathetic denervation and parasympathetic stimulation, we have failed to demonstrate significant decrease in RVSP on cardiac catheterization after application of these interventions. This may be explained by the insufficient follow up period, which is certainly one of the limitations of the present study. The extension of postoperative period to 8–10 months could unmask the effect of anti-remodeling intervention on PRV and RVSP.

## 5. Conclusions

The results of this study have demonstrated the key role of autonomic nervous system in the development and progression of CTEPH. Sympathetic denervation with stellate ganglion ablation, as well as pyridostigmine-induced parasympathetic stimulation, resulted in attenuated vascular remodeling. These salutary changes were associated with altered MMP2 and TIMP1 expression in the lung and decreased FGFb level in the blood. Unilateral vagotomy had no effect on physiological and morphological outcomes of the study. It is evident that there are multiple molecular pathways involved in the mediation of the effects of autonomic nervous system on pulmonary vasculature both in physiological and pathological states. The data obtained may aid in the identification of new therapeutic targets for CTEPH treatment.

## Figures and Tables

**Table 1:** Primers for reverse polymerase chain reaction for assessment of fibrotic gene expression.

Primer	Direct Primer Sequence	Reverse Primer Sequence
Vim	TGCCAACCGGAACAACGAT	ACTGCACCTGTCTCCGGTA
MMP2	GGACCTGTCACTCCCGAGAT	TCCGCCAAATAAACCGATCCT
MMP8	ACCAATGCTGGAGATACGACA	CTGGGAACACGCTTGCTATG
TIMP1	AGACACGCTAGAGCAGATACC	GGTCCGAGTTGCAGAAAGC
TGFβ	AGCGAAGCGACGAGGAGTA	ACTGGGCAGACAGTTTCGG
GAPD	CGGTGTGAACGGATTTGGC	TTGAGGTCAATGAAGGGGTCG

**Table 2:** Echocardiographic parameters 6 weeks after the last administration of microspheres.

Parameter	INT	CTEPH	CTEPH + SD	CTEPH + VD	CTEPH + PS
PA diameter, mm	2.91 ± 0.15	2.91 ± 0.14	3.30 ± 0.31	2.92 ± 0.24	2.96 ± 0.10
RVOT diameter (mm)	3.94 ± 0.17	3.96 ± 0.14	4.07 ± 0.18	4.03 ± 0.35	4.13 ± 0.35
FS LV (%)	52.0 ± 3.8	51.9 ± 12.1	44.5 ± 7.5	51.6 ± 9.2	47.2 ± 6.4
TAPSE (mm)	2.50 ± 0.12	2.55 ± 0.23	2.94 ± 0.61	3.00 ± 0.37	2.51 ± 0.35

INT—intact animals (n = 13), CTEPH—chronic thromboembolic pulmonary hypertension (n = 12), CTEPH + SD—CTEPH + sympathetic denervation (n = 8), CTEPH + VD—CTEPH + vagus denervation (n = 6), CTEPH + PS—CTEPH + pyridostigmine (n = 6), PA—pulmonary artery, RVOT—right ventricle outflow tract, FS LV—fractional shortening of left ventricle, TAPSE—tricuspid annular plane systolic excursion.

**Table 3:** Invasive hemodynamic parameters result six weeks follow up after last microspheres administration.

Parameter	INT	CTEPH	CTEPH + SD	CTEPH + VD	CTEPH + PS
Mean BP, mm Hg	51.3 ± 7.6	56.8 ± 14.1	55.0 ± 22.8	54.4 ± 3.5	58.1 ± 20.9
CO, mL/min	35.4 ± 7.4	49.4 ± 14.4	44.2 ± 12.6	41.6 ± 18.1	49.0 ± 23.5
RVSP/CO ratio	0.48 ± 0.13	0.49 ± 0.15	0.55 ± 0.20	0.53 ± 0.25	0.50 ± 0.14
Heart rate	214 ± 31	255 ± 39	302 ± 78	253 ± 33	207 ± 54

INT—intact animals (n = 13), CTEPH—chronic thromboembolic pulmonary hypertension (n = 11), CTEPH + SD—CTEPH + sympathetic denervation (n = 8), CTEPH + VD—CTEPH + vagus denervation (n = 5), CTEPH + PS—CTEPH + pyridostigmine (n = 5), mean BP—mean blood pressure. RVSP—right ventricle systolic blood pressure, CO—cardiac output.

## Data Availability

Not applicable.
